# Cyclophosphamide-Induced Hemorrhagic Myocarditis

**DOI:** 10.1016/j.jaccas.2025.106613

**Published:** 2026-01-21

**Authors:** Rade R. Jibawi Rivera, David M. Harmon, Joerg Herrmann, Patricia A. Pellikka

**Affiliations:** a Deparment of Internal Medicine, Mayo Clinic, Rochester, Minnesota, USA; b Department of Cardiovascular Medicine, Mayo Clinic, Rochester, Minnesota, USA

**Keywords:** acute heart failure, cancer, cardiac magnetic resonance, cardiomyopathy, echocardiography, imaging, left ventricle, peripheral edema, supraventricular arrhythmias

## Abstract

**Background:**

Cyclophosphamide is a widely used chemotherapeutic agent with a spectrum of toxicities, including a rare drug-related hemorrhagic myocarditis.

**Case Summary:**

A 63 -year-old male with myelofibrosis underwent haploidentical stem cell transplantation with 95 mg/kg cyclophosphamide conditioning. He developed tachycardia, lower-extremity edema, and pulmonary congestion 8 days later. Troponin-T was 512 ng/L, and N-terminal pro-brain natriuretic peptide was 20,142 pg/mL, each previously normal. Electrocardiogram showed low-voltage QRS complexes in limb leads. Echocardiography demonstrated significant left ventricular wall thickening (17 mm), preserved left ventricular ejection fraction (54%), and decreased global longitudinal strain (−9%), all previously normal. Cardiac magnetic resonance imaging suggested myocardial edema and inflammation. Left ventricular T2∗ value near 20 ms suggested intramyocardial hemorrhage. Volume overload and supraventricular arrhythmias were managed conservatively.

**Discussion:**

To our knowledge, there are no reported cases of nonfatal cyclophosphamide-induced hemorrhagic myocarditis at a dose <100 mg/kg.

**Take-Home Message:**

An expedited multimodal cardiac imaging approach is warranted when this rare cardiotoxicity is suspected.

## Visual Summary

**Figure:**
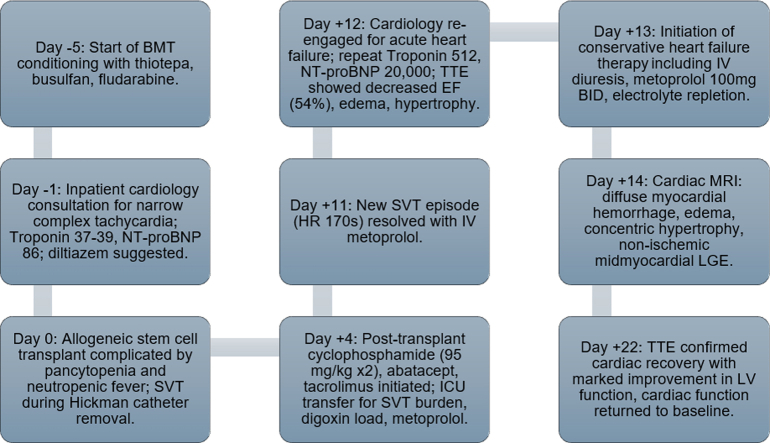

Cyclophosphamide is an alkylating agent that is widely utilized for its immunomodulatory role in the management of malignancies and autoimmune diseases. Along with its immunosuppressive effect, it includes a wide spectrum of toxicities including cardiovascular toxicity.^1^ Although the incidence of cyclophosphamide-induced cardiotoxicity is unknown due to its rarity, studies have reported that left ventricular (LV) dysfunction occurs in over half of patients who undergo transplantation with cyclophosphamide therapy.^1^^,^^2^ The exact mechanism of cardiotoxicity remains unknown; however, translational animal studies have demonstrated increased lipid peroxidation along with a reduction in antioxidant markers.^3^^,^^4^ Toxicity often presents at high doses, typically in the range of 120 to 200 mg/kg, and most reported toxicities highlight a dose-dependency relationship with generalized cardiomyopathy or heart failure.^5^ Given the diagnostic challenge, high-mortality rate, and its potential for rapid deterioration, early recognition at lower medication doses should not be underestimated.^6^ We present a case of cyclophosphamide-related hemorrhagic myocarditis in a patient who underwent low-dose cyclophosphamide conditioning post haploidentical stem cell transplantation (HSCT).

**Take-Home Message**

• An expedited multimodal cardiac imaging approach (echocardiography and cardiac magnetic resonance imaging) is warranted when this rare cardiotoxicity is suspected.

## Case Presentation

**Figure 1:**
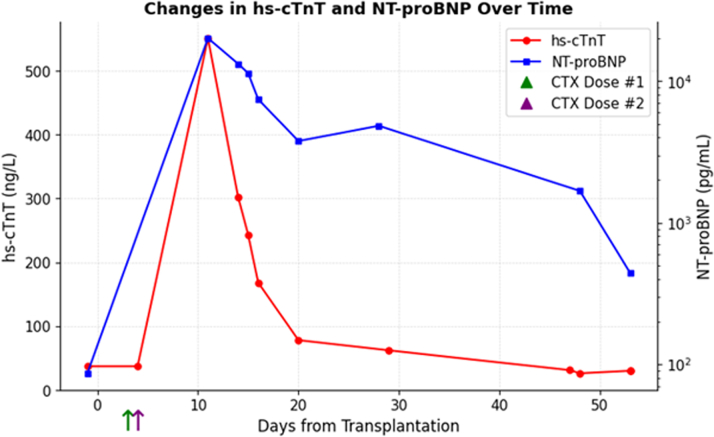
Serial hs-cTNT and NT-proBNP Levels Patent Against Days From Allogeneic Stem Cell Transplantation (Day 0) Cyclophosphamide was administered on day +3 and day +4 as part of the graft versus host disease prophylaxis. A marked rise in hs-cTNT was observed on day +11 with a peak of 512 ng/L. A gradual decline was observed and returned to baseline. In parallel, NT-proBNP increased from a baseline of 86 pg/mL to 20,142 pg/mL, also peaking around day +11. A gradual decline was observed with latest levels around 1,500 pg/mL. These biomarkers were checked to demonstrate the temporal course of rapid onset and resolution of this condition.

**Figure 2:**
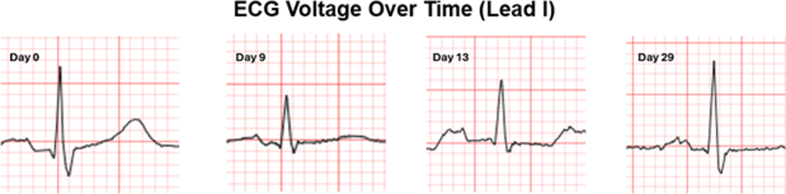
ECG Changes Over Time for Lead I 12-lead ECG was recorded on a standard paper speed of 25 mm/s and a gain of 10 mm/mV. Voltage was recorded as sum of R- and S-wave amplitudes. At baseline (day 0), voltage measured 1.0 mV, decreasing to 0.5 mV by day 9. ECG voltage gradually improved to near baseline by day 29.

**Figure 3:**
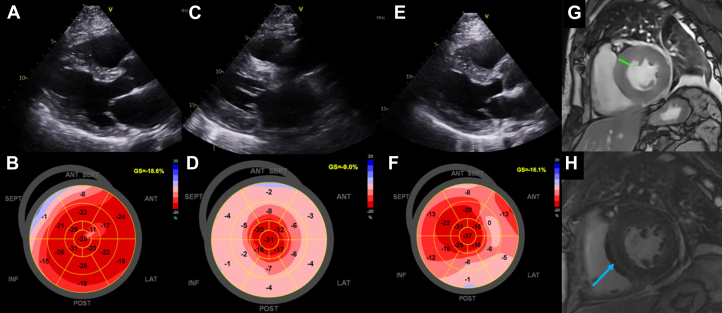
Cardiac Imaging at Baseline and Post-Cyclophosphamide Administration Baseline echocardiogram (TTE) with normal left ventricular (LV) wall thickness (A) and normal global longitudinal strain (B). TTE 8 days after administration of cyclophosphamide with concentric LV wall thickening (C) and reduced global longitudinal strain (D). TTE 18 days after cyclophosphamide administration showing decreased LV wall thickness (E) and improved longitudinal strain (F). Cardiac magnetic resonance imaging on day 8 demonstrated concentric LV myocardial thickening (G; up to 20 mm green line) and nonischemic, mid-myocardial late gadolinium enhancement (H; blue arrow).

A 63 y/o male with past medical history of myelofibrosis, polycythemia vera, neuroendocrine tumor stage IA, portal vein thrombosis, splenectomy, and diastolic hypertension underwent HSCT with cyclophosphamide conditioning for graft vs host disease (GVHD). Prior to HSCT, he underwent cardiology evaluation for supraventricular tachycardia (SVT) in the setting of his malignancy. Diagnostic workup was reassuring with normal cardiac biomarkers (high-sensitivity troponin T and N-terminal pro-brain natriuretic peptide) and unremarkable transthoracic echocardiogram (TTE). Three days after transplantation, the patient received GVHD prophylaxis with cyclophosphamide at 95 mg/kg, along with abatacept and tacrolimus. Seven days after cyclophosphamide administration, the patient developed worsening SVT, lower-extremity edema, and tachypnea. Physical examination revealed inspiratory crackles at the mid and lower lung fields bilaterally, elevated jugular venous pulsation, and bilateral lower-extremity edema. Laboratory evaluation revealed a high-sensitivity troponin T of 512 ng/L (normal <15 ng/L) and N-terminal pro-brain natriuretic peptide at 20,142 pg/mL (normal <88 pg/mL), and a 12-lead electrocardiogram showed new low-voltage QRS complexes in limb leads (Figures 1 and 2). TTE demonstrated significant thickening of the LV walls (17 mm septum; 16 mm posterior wall) with preserved LV ejection fraction (LVEF) at 54% (baseline LVEF: 66%) and significantly reduced global longitudinal strain (GLS; −9%), all of which were previously normal during baseline echocardiogram (Figure 3).

**Visual Summary:**
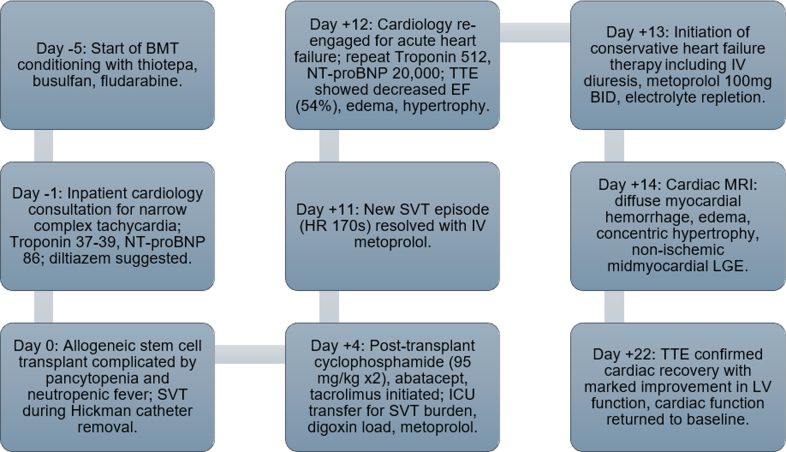
Timeline of Case

With the development of clinical heart failure and subacute increase in LV wall thickness, cardiac magnetic resonance imaging (CMR) was obtained. T2 mapping revealed elevated relaxation times, diffuse nonischemic mid-myocardial low-level late-gadolinium enhancement, consistent with myocardial edema and inflammation, respectively. Global LV T2-star value was near 20 ms, suggesting a hemorrhage within the myocardium supporting a diagnosis of cyclophosphamide cardiotoxicity (Figure 3). Heart failure and SVT were managed conservatively with intravenous diuresis and oral beta-blockade, respectively. Interestingly, the patient developed episodes of hematuria and hemoptysis in the weeks following initial cardiac symptoms alongside severe thrombocytopenia (30 × 10(9)/L).

Two weeks after his initial cardiac symptoms, cardiac biomarkers improved, electrocardiogram limb lead voltage normalized, and cardiac symptomology subsided (Figures 1 and 2). A repeat TTE obtained 18 days after cyclophosphamide administration showed decreasing LV wall thickness and improved global strain (Figure 3). Clinical improvement continued with normalization of LV wall thickness, LVEF, and GLS on 6-month TTE, with normalization of T1 and T2 values at the corresponding 6-month CMR.

## Discussion

Chemotherapy conditioning with cyclophosphamide is a mainstay therapy for GVHD prophylaxis.^7^ Cyclophosphamide-induced cardiotoxicity is a well-known, highly morbid side effect thought to be dose-dependent. Prior studies and case reports commonly reported cardiotoxicity with high doses (120-200 mg/kg) of cyclophosphamide and cardiac events occurring within 3 months of treatment administration.^5^ Similarly, these prior cases reported no acute changes in LVEF.^1^

Our patient experienced cyclophosphamide-related hemorrhagic myocarditis with a lower-dose cyclophosphamide protocol at 95 mg/kg post HSCT with rapid onset and improvement of cardiac symptoms course of 3 weeks. His total cyclophosphamide dose of 95 mg/kg was considered 20% of the lower limit for “high-dose” therapy. Following the development of cardiac symptoms 8 days after medication administration, the patient experienced a significant decrease in LVEF of 12% and GLS on TTE as well as magnetic resonance imaging–supported myocardial edema, inflammation, and hemorrhage. Of note, his vascular injury leading to myocardial hemorrhage, hematuria, and hemoptysis may have been potentiated by the profound thrombocytopenia. To our knowledge, there are no reported cases of nonfatal cyclophosphamide-induced hemorrhagic myocarditis at a dose of <100 mg/kg in the literature.^8^^,^^9^

Current cardio-oncology guidelines do not offer direct management recommendations for patients undergoing HSCT and GVHD prophylaxis with cyclophosphamide. Existing recommendations focus on anthracycline-induced cardiotoxicity with prophylaxis use of angiotensin-converting enzyme-inhibitors, angiotensin receptor blockers, and beta-blockers to prevent decline in LVEF.^10^^,^^11^ In the absence of guideline-direct recommendations, our management focused on conservative measures tailored to the patient's symptomology and biomarker profile. Within 18 days, his symptoms improved substantially with evidence of cardiac function returning to baseline on TTE. Clinically he continued to improve with conservative management at long-term (6-month) follow-up.

This case underscores the critical need for early multimodality imaging when cyclophosphamide cardiotoxicity is suspected. CMR offers immense diagnostic value in this case, particularly with the ability to evaluate for myocardial edema as a result of myocardial hemorrhage. Until specific recommendations are developed, management for this drug-related cardiotoxicity remains supportive with an emphasis on early detection with the use of noninvasive diagnostics (ie, cardiac biomarkers and CMR).

## Conclusions

Cyclophosphamide cardiotoxicity should be considered even with reduced dose (<100 mg/kg) protocols. Echocardiography on day 18 post cyclophosphamide dose administration showed decreasing LV wall thickness and improving global strain, with decreasing cardiac biomarkers correlating with the patient's clinical improvement.

## Funding Support and Author Disclosures

The authors have reported that they have no relationships relevant to the contents of this paper to disclose.
